# Advances in gene editing for legume improvement: technologies, progress, and prospects

**DOI:** 10.3389/fgeed.2026.1789952

**Published:** 2026-04-15

**Authors:** Shallu Thakur, Shalini Pareek, G. P. Dixit, Geoffrey Meru, Alok Das

**Affiliations:** 1 Tropical Research and Education Center, Horticultural Sciences Department, University of Florida, IFAS, Homestead, FL, United States; 2 ICAR- Indian Institute of Pulses Research, Kanpur, India

**Keywords:** CRISPR/Cas9, gene editing, GRNA, legume, TALEN, ZFN

## Abstract

Legumes are among the most important protein-rich crops in global agri-food systems. To meet the rising protein demand of a growing population, significant efforts are underway to enhance legume yield, nutritional quality, and resilience to environmental stresses through the manipulation of key genetic traits. Advanced technologies-including genetic engineering, gene editing, genomic selection, next-generation sequencing, single-cell genomics, and multi-omics-are accelerating legume improvement due to their high precision and efficiency. This review focuses on major gene-editing technologies, namely, CRISPR/Cas9 (Clustered Regularly Interspaced Short Palindromic Repeats/CRISPR-associated protein 9), TALENs (Transcription Activator-Like Effector Nucleases), ZFNs (Zinc Finger Nucleases), base editing (BE), and prime editing (PE), and their applications in key legume crops such as soybean (*Glycine max*), cowpea (*Vigna unguiculata*), chickpea (*Cicer arietinum*), groundnut (*Arachis hypogaea*), pea (*Pisum sativum*), barrel clover (*Medicago truncatula*), alfalfa (*Medicago sativa*), and *Lotus japonicus*. Among these platforms, CRISPR/Cas9 is the most widely adopted in legumes, largely due to its simplicity, versatility, and dependence on accurate genome sequence information and guide RNA (gRNA) design. Advances in next-generation sequencing and the growing availability of intuitive online gRNA design tools have streamlined CRISPR workflows, improving accessibility and precision. The present review indicates that CRISPR-P is the most used gRNA design tool in legume research, likely due to its early development for plant systems and integrated off-target prediction features. Therefore, alongside reviewing gene-editing applications, we emphasized the critical role of robust gRNA design tools as a foundation for successful genome editing. Future integration of artificial intelligence and large language models is expected to further enhance target prediction accuracy, minimize off-target effects, and enable more precise genome-editing strategies in legumes.

## 1 Introduction

Legumes are the third largest family of angiosperms, encompassing more than 19,500 known species across 700 genera, and include a diverse range of economically and nutritionally important food crops (Lewis et al., 2005). They are the important source of plant-based proteins and essential amino acids (Bhowmik et al., 2021). This includes both grain legumes (*Glycine max, Vigna unguiculata, Cicer arietinum, Arachis hypogaea*, *Pisum sativum*, *Cajanus cajan*) and non-grain legumes (*Medicago truncatula, Medicago sativa and Lotus japonicus)* (Thakur et al., 2021; Baloglu et al., 2022). In addition, legumes are the important biological source of nitrogen fixation in the soil, which enhances agricultural productivity (Wang et al., 2017; Jain et al., 2023). Pulses are the dry edible seeds of grain legumes, harvested for human consumption. In recognition of their nutritional and agricultural value, the United Nations declared 2016 the “International Year of Pulses”. World Pulses Day, established by the United Nations General Assembly (2019), is observed annually on 10 February to raise awareness about the role of pulses in nutrition, food security, sustainable agriculture, and climate resilience. Despite many health benefits and ecological significance, legume productivity remains constrained by a range of abiotic and biotic stresses, underscoring the urgent need for genetic gain in yield, nutritional quality, and stress tolerance (Kumari et al., 2025).

Conventional breeding has contributed significantly to the legume improvement, but its progress is often constrained by long generation cycles, limited genetic variability, and complex polygenic traits (Afzal et al., 2020; Jain et al., 2023; Mousavi-Derazmahalleh et al., 2019; Varshney et al., 2018; Varshney et al., 2019). These challenges restrict the speed and precision of cultivar development. To overcome these limitations, molecular breeding approaches have been increasingly integrated into legume improvement programs. Over the past decade, advances in legume genomics have transformed breeding strategies. More than 35 legume species have been sequenced, and their genomic resources are now publicly available, facilitating the discovery of molecular markers and enabling marker-assisted selection (MAS) and genomic-selection (GS) for complex traits (Varshney et al., 2019). Building on these genomic advances, genomics-assisted breeding approaches have emerged as valuable complements to conventional breeding, improving the efficiency and precision of selecting desirable agronomic traits (Mousavi-Derazmahalleh et al., 2019).

Over the last decade, genome editing technologies have emerged as powerful and versatile tool, for introducing targeted genetic modifications at specific genomic loci with unprecedented accuracy (Arora and Narula, 2017). Several genome editing platforms, such as Zinc-Finger Nucleases (ZFNs), Transcription Activator-Like Effector Nucleases (TALENs), and Clustered Regularly Interspaced Short Palindromic Repeats/CRISPR-associated protein 9 (CRISPR/Cas9)-based systems, have been successfully deployed in legumes (Curtin et al., 2011; Du et al., 2016; Kumari et al., 2025). Emerging new genome editing technologies such as base editing (BE) and prime editing (PE) have further expanded the possibilities for precise nucleotide changes without introducing double strand breaks (DSBs) (Komor et al., 2016; Mishra et al., 2020; Zhong et al., 2022). Collectively, these tools have been applied across major legume species including *G*. *max*, *V*. *unguiculata*, *C*. *arietinum*, *A*. *hypogaea*, *P*. *sativum*, *C*. *cajan*, *M*. *truncatula*, *M. sativa*, and *L*. *japonicus*, enabling significant advancements in disease resistance, abiotic stress tolerance, nutritional enhancement, and yield improvement (Bhowmik et al., 2021; Bhowmik et al., 2023; Wolabu et al., 2024; Fukuda et al., 2024; Niu et al., 2024; Abdallah et al., 2025; Asiamah et al., 2025).

Till now, CRISPR/Cas9 has become the most widely used technology due to its simplicity, programmability, and high editing efficiency (El-Mounadi et al., 2020; Thakur et al., 2025). A key determinant of successful CRISPR/Cas9 editing, is the design of an efficient and specific guide RNA (gRNA), which directly governs editing precision and off-target activity (Liang et al., 2016). The availability of numerous online gRNA design tools has simplified this step, improving both accessibility and accuracy in genome editing workflows. Among these tools, CRISPR-P has emerged as the most widely utilized tool in plant and legume research, owing to its early development for plant genomes and its integrated off-target prediction features (Minkenberg et al., 2019). Beyond technical considerations, regulatory framework for genome-edited crops plays a pivotal role in determining their field deployment and commercialization (Asiamah et al., 2025). Regulatory landscape varies substantially across countries, influencing research translation, public acceptance, and global trade (Rose et al., 2020; Asiamah et al., 2025; Thakur et al., 2025). Therefore, understanding the evolving policy landscape is essential when evaluating the broader impact of genome editing in legumes.

Given the rapid expansion and diverse applications of genome editing platforms in legume, there is a growing need to synthesize current knowledge and evaluate the key factors, that underpin successful editing outcomes, including the availability of genome sequence information, functional understanding of genes and their roles in biochemical pathways, and the selection of appropriate gRNA design tools, all of which directly influence editing precision and efficiency. Therefore, this review brings together recent advances in genome editing across major legume species while also assessing the design tools that support CRISPR workflows. We highlight technological developments, emerging approaches (BE and PE), and key applications of genome editing platforms in legumes, and provide a critical evaluation of commonly used gRNA design tools, emphasizing their role as an essential prerequisite for achieving accurate and efficient genome modification and briefly examines regulatory landscape shaping the adoption of genome-edited legumes. Overall, this review serves as a comprehensive reference on legume genome-editing efforts and an integrated resource for future research and legume improvement initiatives.

## 2 Genome editing technologies in legumes

Several genome editing technologies have been used in legumes including ZFNs, TALENs, CRISPR/Cas9, BE and PE. However, apart from CRISPR/Cas9, reports of successful genome editing using other platforms remain relatively limited. In this section, we provide a brief overview of these genome-editing technologies, with particular emphasis on their application and relevance in legumes.

### 2.1 Zinc finger nuclease (ZFN)

**FIGURE 1:**
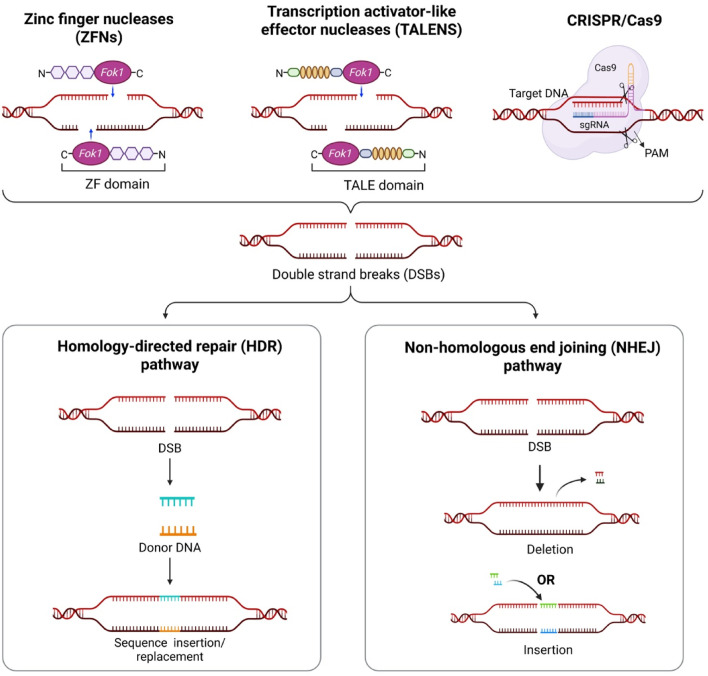
Genome editing nucleases (ZFNs, TALENs, CRISPR/Cas9) introduce double strand breaks (DSBs) at target DNA sites. The resulting breaks are repaired by non-homologous end joining (NHEJ), producing mutations, or by homology-directed repair (HDR), enabling precise editing.

In the 1900s, the first targeted genome editing tool known as ZFNs were developed, after extensive understanding of DNA repair mechanisms. ZFNs are considered as *first generation* of site-specific nucleases (SSNs), where zinc finger (ZF) domain is merged with nuclease domain (*Fok*I restriction enzyme) for the DNA cleavage (Osakabe and Osakabe, 2015) (Figure 1). Together they are used in pairs to create DSBs. This cleavage induces cellular repair process resulting in efficient tailoring of the targeted locus of DNA. ZF domain consists of up to 6 proteins and binds to the DNA target. Zn^+2^ ions provide stability to the engineered Cys2–His2 residues of ZFs and each of these proteins interacts with three base pairs (Urnov et al., 2010). The major drawback of ZFNs compared with other genome-editing technologies is the considerable time and expertise required for their design. Constructing and optimizing ZF arrays is technically challenging, and only a limited number of well-characterized ZF pairs are currently available (Baloglu et al., 2022). In legumes, ZFN based genome editing has been reported in soybean (*G*. *max*). Curtin et al. (2011) targeted *DICER*-like genes (*DCL4a/b* and *DCLb)* and other RNA-silencing–related genes using ZFNs, achieving efficient mutations in seven of the nine targets. These results demonstrate the effectiveness of ZFNs for generating mutations in duplicated genes. However, relatively few ZFN studies exist in legumes because the technology is constrained by limited target-site availability and lower DNA-binding affinity (Gupta and Musunuru, 2014).

### 2.2 Transcription activator-like effector nucleases (TALENs)

TALENs, as a second-generation gene editing tool, comprises the *Fok*I nuclease domain and TALE protein, containing customizable DNA-binding repeats. TALE proteins are derived from pathogenic *Xanthomonas* bacteria that translocate TALEs proteins into host cell cytoplasm. To enhance the bacterial colonization and infection, TALs work as host’s transcription machinery and make developmental changes in plant beneficial to infection (Boch et al., 2009). Structurally, TALEs contain a central domain of tandem repeats (33–35 amino acid long chain responsible for DNA binding) and a transcription activator domain (Figure 1). The hypervariable residues present at the 12th and 13th position (also called Repeat Variable Di-Residues) of these tandem repeats decides the specific binding with target DNA. In TALE, the transcription activator domain is replaced with nuclease *Fok*I which forms TALEN. To achieve DSBs via *Fok*I, TALENs are used in pairs. Each pair binds to opposite strand of target DNA and is separated by spacer domain (Zhang et al., 2014). The application of TALENs in legumes has been demonstrated primarily in *M. truncatula*, *L*. *japonicus*, groundnut and soybean. In *L*. *japonicus*, TALENs have been applied to functional genomics studies to improve the understanding of genes involved in indeterminate nodule development (Wang et al., 2016). In *M*. *truncatula*, the efficacy of editing system has been reported using gene knockout (*MtHen*1) strategy (Curtin et al., 2018). In groundnut, TALEN-mediated editing of the *AhFAD2* gene successfully enhanced seed oil quality by increasing oleic acid content (Wen et al., 2018). In soybean, TALENs have been used to target several genes, including *DCL* (*Dicer-like2*), *GmPDS11* and *GmPDS18* resulting in albino and dwarf phenotypes (Du et al., 2016; Curtin et al., 2018). Additionally, *GmDcl2b* was also targeted, resulting in heritable combinatorial mutations, further demonstrating the utility of TALENs in soybean functional genomics (Curtin et al., 2017; Curtin et al., 2018; Du et al., 2016). TALEN-mediated editing of the fatty acid biosynthesis genes *FAD2-1 A/B* and *FAD3A* has been reported to improve seed quality by increasing oleic acid and decreasing linolenic acid, respectively (Haun et al., 2014; Demorest et al., 2016). Knockout of these genes led to a significant reduction in polyunsaturated fats and an increase in healthier monounsaturated oleic acid. This was the basis for the first commercialized gene-edited plant product (Calyno) in the USA. Despite these successes, the broader use of TALENs in legume remains limited due to several practical constraints. A major disadvantage is the relatively large size of the TALEN coding sequence (∼3 kb), which complicates delivery compared to ZFNs, whose cDNA is approximately 1 kb. Moreover, the highly repetitive structure of TALENs can interfere with cloning stability and may impede efficient packaging into viral vectors, further restricting their utility in certain delivery platforms (Gupta and Musunuru, 2014). Another limitation of TALENs is the difficulty of multiplex genome editing, since each target requires custom DNA-binding domains and co-delivery of multiple TALEN pairs (Malzahn et al., 2017).

### 2.3 CRISPR/Cas9

CRISPR is short, palindromic repeated DNA sequences found in the genomes of prokaryotes. Together with Cas9 protein, the CRISPR system functions as an adaptive immunity in prokaryotes to defend themselves against viruses or bacteriophages (Hille and Charpentier, 2016). In the following section, we discuss in detail about this technique and its applications in legume crops.

#### 2.3.1 CRISPR/Cas9 components

**FIGURE 2:**
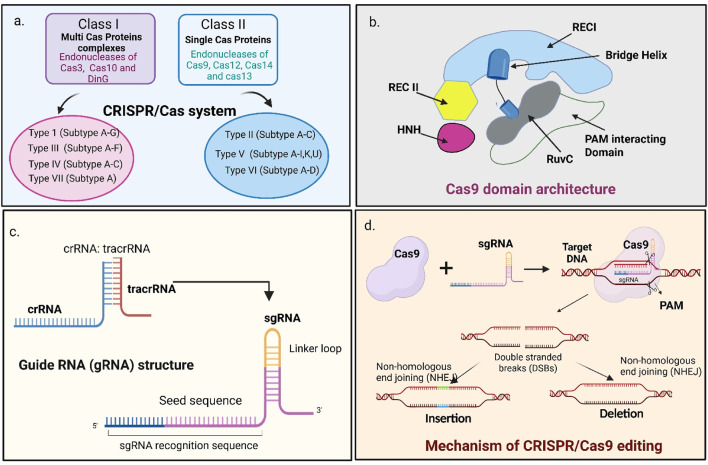
Overview of CRISPR/Cas9 system classification, structural components, and gene-editing mechanism. **(a)** Classification of CRISPR/Cas systems. **(b)** Domain architecture of *Streptococcus pyogenes* Cas9 (SpCas9), showing the Recognition (REC) lobe and the Nuclease lobe (RuvC, HNH, and Protospacer Adjacent Motif (PAM)-interacting domains). **(c)** Structure of natural crRNA and tracrRNA and their engineered fusion into a single guide RNA (gRNA) used for genome editing. **(d)** Mechanism of CRISPR/Cas9-mediated DNA cleavage: the Cas9–sgRNA complex recognizes a PAM sequence, binds to the complementary genomic target, and introduces a DSB via HNH and RuvC nuclease domains, followed by repair through NHEJ to generate indels.

The CRISPR/Cas system is grouped into two classes based on the organization of their effector proteins. Class I systems (Types I, III, IV, VII) use multi-subunit effector complexes, whereas Class II CRISPR systems (Types II, V, and VI) utilize a single, multidomain crRNA-guided effector protein such as Cas9, Cas12 that performs the functions carried out by the multi-subunit effector complexes of Class I systems (Liu Z. et al., 2020; Makarova et al., 2025) (Figure 2a). To date, seven CRISPR–Cas types and 46 subtypes have been identified (Koonin et al., 2017; Makarova et al., 2020; Makarova et al., 2025). Among these, Types I, II and III are the most extensively studied. Interestingly, the same organism can possess all three of these systems simultaneously. Type I systems use the signature nuclease–helicase Cas3 to degrade target DNA. Type II systems, characterized by the Cas9 nuclease with its RuvC and HNH domains, create targeted DSBs (Gasiunas et al., 2012; Jinek et al., 2012). Type V systems employ Cas12, which contains only a RuvC domain, while Type VI systems use Cas13, an RNA-targeting nuclease. The newly identified Type VII CRISPR systems rely on Cas14 for crRNA processing, with its ribonuclease activity dependent on the Cas5–Cas7 complex (Makarova et al., 2025). Among all, CRISPR/Cas9 type II system is the most popular due to its adaptability and advanced gene editing capabilities (Liu Z. et al., 2020).

Despite this diversity, most CRISPR systems rely on two core components, a gRNA and an associated Cas effector protein that together direct sequence-specific cleavage.

##### 2.3.1.1 Cas9

Cas9 was first isolated from *Streptococcus pyogenes* (SpCas9). It is a 1,368-amino-acid, multidomain DNA endonuclease that cleaves target DNA, generating DSBs (Mei et al., 2016). Cas9 protein contains 2 lobes, one is recognition lobe (REC), and another is nuclease lobe (NUC). REC lobe contains two domains called REC1 and REC2 that bind to RNA. NUC lobe has three domains, namely, RuvC, HNH, and Protospacer Adjacent Motif (PAM) interacting domain (PID). RuvC and HNH domains cut each of the single stranded DNA and PAM interacting domains confirm the PAM specificity and initiate binding to the target DNA (Nishimasu et al., 2014) (Figure 2b).

##### 2.3.1.2 Guide RNA (gRNA)

The gRNA is composed of two components: CRISPR RNA (crRNA) and trans-activating CRISPR RNA (tracrRNA). The crRNA contains an approximately 18–20 nucleotide sequence that is complementary to the target DNA and directs sequence-specific binding. The tracrRNA forms a structural scaffold that binds to Cas9, stabilizing the complex and enabling the nuclease to cleave the target DNA. For gene editing applications, gRNA is designed by combining crRNA and tracrRNA to form a gRNA (Mei et al., 2016) (Figure 2c).

#### 2.3.2 CRISPR/Cas9 working mechanism

The mechanism of CRISPR/Cas9 includes three steps: target recognition, cleavage and repair (Shao et al., 2016). The designed gRNA, containing the 5'crRNA component, direct Cas9 to the complementary target sequence. The *Sp*Cas9 protein introduces DSBs, often three base pairs upstream of the PAM region, a short, conserved DNA sequence located downstream of the target site. The most used PAM sequence is 5'- NGG-3' (Ceasar et al., 2016). After binding to the PAM, Cas9 unbinds DNA and forms a DNA-RNA hybrid and activates DNA cleavage. HNH nuclease domain cleaves the DNA strand complementary to the gRNA, while RuvC domain cleaves the non-complementary strand, resulting in a blunt ended DSBs. Finally, the host cell machinery repairs the break using non-homologous end joining (NHEJ), or the homology-directed repair (HDR) pathway when a suitable repair template is available (Jiang and Doudna, 2017; Asmamaw and Zawdie, 2021). NHEJ repairs the break by directly joining the DNA ends without using a repair template and operates throughout the cell cycle. Although it is the most common and efficient repair mechanism. It is error-prone and often results in small insertions or deletions (indels) that may cause frameshift mutations or premature stop codons (Yang et al., 2020). In contrast, HDR is a more precise repair pathway that requires a homologous DNA template and is most active during the late S and G2 phases of the cell cycle. In genome editing, HDR enables accurate gene insertion or replacement by using an external donor DNA template that shares sequence similarity with the target region (Liu et al., 2019; Yang et al., 2020) (Figures 1, 2d).

#### 2.3.3 Guide RNA (gRNA) designing workflow

**FIGURE 3:**
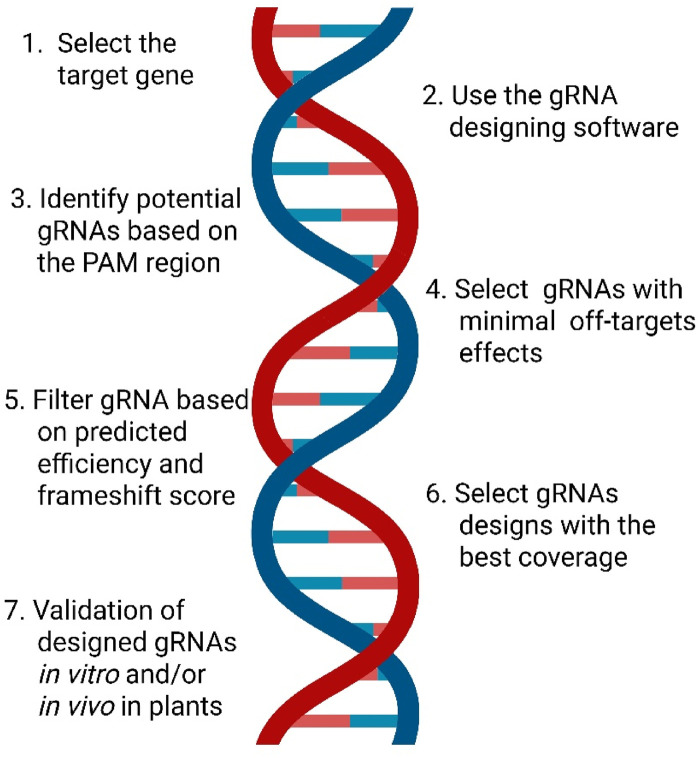
Overview of a simple strategy for designing gRNAs to generate CRISPR/Cas9 knockouts, including target selection, guide design with minimal off-target effects.

Targeting efficiency of the *Sp*Cas9 nuclease in the CRISPR/Cas9 system primarily depends on the designed gRNA and the presence of an appropriate PAM near the target site (Wright et al., 2016). In plants, large gene families and repetitive sequences increase the risk of off-target binding, which can lead to unintended and unpredictable effects. Several online tools have been developed to aid in the design and evaluation of gRNAs, including CHOP-CHOP, CRISPR-P 2.0, CRISPR-PLANT, Benchling, sgRNAcas9, CRISPR Inc., CRISPRdirect, Cas-OFFinder, E-CRISP v5.4, CRISPOR, and CRISPseek (Concordet and Haeussler, 2018; Liu et al., 2017; Labun et al., 2019; Thakur et al., 2021). The differences among these tools and their relevance for legume genome editing are summarized in Table 1, while the key limitations of gRNA prediction and design tools are outlined in Supplementary Table S1. A simplified workflow for designing an effective gRNA is illustrated in Figure 3.

**TABLE 1:** Key differences among widely used gRNA design tools.

Tool	Primary focus	Off-target prediction	Genome support	Plant/Legume usefulness	Primer/Validation tools	Sources
CHOPCHOP	sgRNA/TALEN design, visualization	Yes, with Bowtie	Broad (plants incl. *Arabidopsis*)	High; supports plant genomes, primers for validation	Yes, automatic primers/restriction sites	https://chopchop.cbu.uib.no
CRISPR-P 2.0	Plant sgRNA design	Improved scoring	49 plant genomes	Highest for legumes; used in legume CRISPR projects	Limited details	http://crispr.hzau.edu.cn/CRISPR2/
CRISPR-PLANT	Plant-specific editing (limited info)	Unknown	Plants	Moderate; plant-focused but sparse data	Unknown	http://omap.org/crispr/
Benchling	Batch gRNA design, assembly	On/off-target scores	Custom upload	High; flexible for any genome incl. legumes	Plasmid assembly	https://www.benchling.com/crispr
sgRNAcas9	sgRNA design package	Minimized off targets	Any *via* FASTA	Moderate; software for custom genomes	Vector design	Xie et al. (2014)
CRISPRdirect	Highly specific gRNAs	Yes, multi-species	350+ species, custom DB	High; add legume genomes easily	No	https://cscjp.co.jp/item/crisprdirect_en.html
Cas-OFFinder	Off-target search	Advanced, unlimited mismatches	Any genome	High as companion tool for plants	No	Bae et al. (2014)
E-CRISP v5.4	Target site ID, libraries	Yes	Broad	Moderate; flexible for plants	Experiment-oriented	Heigwer et al. (2014)
CRISPOR	Guide selection, cloning	Multiple scores	Many genomes	High; non-model organisms, primers	Yes, cloning/validation primers	Concordet and Haeussler (2018)
CRISPseek	gRNA ID (bioconductor)	Cleavage scores	Any, R-based	Moderate; programmable for legumes	Filtering options	Zhu et al. (2014)

According to Mohr et al. (2016) general considerations for designing an appropriate gRNA for making CRISPR knockouts via NHEJ are as follows: 1) target a gene-specific region, 2) target the exon region of the gene, 3) target the gRNAs with minimal off targets, 4) filter with a predicted efficiency score, 5) avoid promoter terminator sequence, and 6) avoid restriction sites used for cloning the gRNA.

**FIGURE 4:**
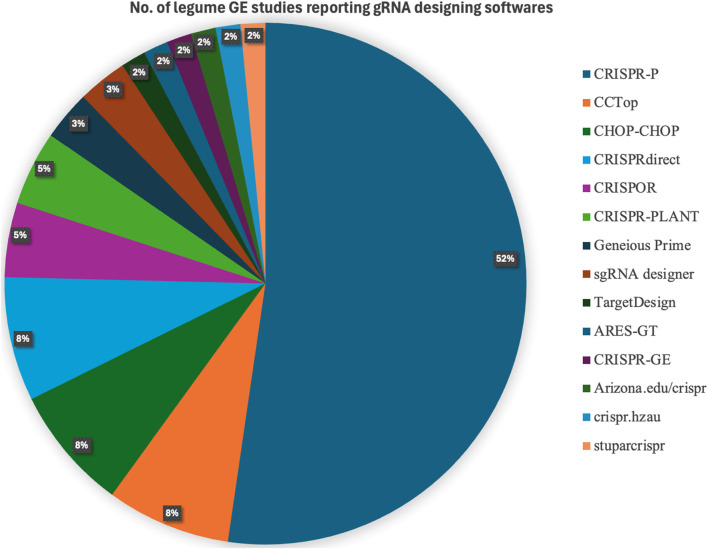
Pie chart illustrates the distribution and relative frequency of different gRNA design software tools used in CRISPR/Cas9 based gene editing studies in legumes. It highlights the proportion of research efforts relying on various online platforms and bioinformatics tools for gRNA designing.

To identify commonly used gRNA design tools in legume genome-editing studies, we conducted a manual survey of published reports summarized in Table 2. Only studies that explicitly specified the software used for gRNA design were included in the analysis. Based on the reported tools in these studies, CRISPR-P was the most frequently used (53%), followed by CHOPCHOP, CCTop, and CRISPRdirect, each accounting for approximately 8% of usage (Figure 4). The widespread adoption of CRISPR-P likely reflects its plant-specific genome databases, user friendly interface, and ability to predict targets while assessing potential off-target effects, that are particularly valuable for legume research where species-specific genome references improve guide design accuracy (Liu et al., 2017). However, CRISPR-P relies largely on mismatch-based off-target prediction and requires well-annotated genomes, highlighting the need for complementary tools to improve predictive accuracy, especially for species with complex or incomplete genome assemblies (Liu et al., 2017). More broadly, currently available gRNA design tools have distinct limitations related to off-target prediction accuracy, genome coverage, scoring systems, and lack of chromatin context consideration (Heigwer et al., 2014; Naito et al., 2015). For example, CHOPCHOP and CRISPRdirect rely primarily on mismatch-based filtering, while CRISPR-P and CRISPR-PLANT are limited to well-characterized plant genomes (Labun et al., 2019; Haeussler et al., 2016; Lei et al., 2014). In contrast, Cas-OFFinder focuses solely on off-target site identification and does not predict guide efficiency (Bae et al., 2014; Tycko et al., 2019). A summary of these limitations is provided in Supplementary Table S1.

**TABLE 2:** CRISPR/Cas9 applications in legumes.

Legume	Target gene	CRISPR/Cas9 mediated strategy	Target gene function	gRNA designing software	Result	Reference
*Medicago sativa* L	*MsC3H*	Knockout	Lignin biosynthesis	CRISPR-P	Reduced lignin deposition and improved forage quality	Wolabu et al. (2024)
	*MsFTa1*	Knockout	Floral integrator and activator gene	CRISPR-P	Higher forage biomass yield	Wolabu et al. (2023)
	*MsSGR*	Used different promoters to drive Cas9 expression	Chlorophyll-degrading Mg^++^-dechelatase	CRISPR-P	Strong, mild and weak mutants	Wolabu et al. (2020)
*Medicago truncatula*	*MtLYK10*	Knockout	Nodule symbiosis	CRISPR-P 2.0 and targetDesign	Delayed nodulation in mutant plants	Zhang C. et al., 2024
	*MtZPT2-2*	Knockout	Negatively regulates salt tolerance	-	Increased Na^+^ accumulation	Huang et al. (2024)
	*ChOMT1*	Gene disruption	Produce 4,4 -dihydroxy-2 -methoxychalcone (DHMC)	CRISPR-P 2.0	Decreased DMHC and bioreporter luminescence in rhizospheres	Wu et al. (2024b)
	*MtSOC1*	Knockout	Promotes flowering	Geneious prime software v 2019.1.1	No flowering	Poulet et al. (2024)
	*MtHHO3*	Loss-of-function	Negatively regulates salt tolerance	-	Resistant to salt stress	Wang et al. (2024b)
	*NCR086* and *NCR314*	Knockout	Involved in differentiation of rhizobia	CRISPOR	Ineffective symbiotic phenotype	Saifi et al. (2024)
	*PALM1*	Knockout	Encodes a Cys(2)His(2) zinc finger transcription factor	CRISPOR	Multifoliate phenotype	Zhao et al. (2024)
	*NCR068, NCR089, NCR128* and *NCR161*	Targeted mutagenesis (InDel)	Control terminal differentiation of endosymbiotic rhizobia in nodules	CRISPOR	NCR peptides are not essential for symbiosis	Güngör et al. (2023)
	*MtSUP*	Targeted loss-of-function	Regulate floral organ number	ARES-GT software	Multi-flower phenotype	Rodas et al. (2021)
	*CYP93E2* and *CYP72A61*	Gene disruption by indels	Soyasapogenol B biosynthesis	CRISPR-P	51 *cyp93e2* mutant plant lines	Confalonieri et al. (2021)
	*MtPDS*	Knockout	Codes for phytoene dehydrogenase /Chromoplastic protein	-	Plant obtained with 70% mutation efficiency	Wolabu et al. (2020)
	*MtPDS*	Knockout	Codes for phytoene dehydrogenase /Chromoplastic protein	-	Homozygous and biallelic mutants	Zhang et al. (2020)
	*NPD* genes	Multiplex genome editing	Responsible for nodulation	-	Smaller and ineffective nodules, earlier onset of nodule senescence	Trujillo et al. (2019)
	*MtHen1*	Gene disruption by indels	Encodes for *Hua enhancer1* gene	Broad Institute’s sgRNA designer	Efficient mutation	Curtin et al. (2018)
	*PHO2-*like*, PNO1-*like, and *PEN3-*like	Knockout (frame-shift mutations)	Responsible for rooting and nodulation	Broad Institute’s sgRNA designer v.1	Decreased nodule production	Curtin et al. (2017)
	*MtPDS*	Targeted mutagenesis	Codes for phytoene dehydrogenase /Chromoplastic protein	-	Albino plants	Meng et al. (2017)
	*GmGS1, GmCHI20, MtGUS*	Targeted mutagenesis	-	http://stuparcrispr.cfans umn.edu/CRISPR	Mutated genes	Michno et al. (2015)
*Pisum sativum*	*PsBAS1*	Knockout	Enzyme involved is saponin biosynthesis	CCTop	99.8% reduction of saponins in their seeds	Hodgins et al. (2024)
	*LOX*	Knockout	Generate specific volatile organic compounds	CCTop	Improved aroma and fatty acid profiles	Bhowmik et al. (2023)
	*PsPDS*	Knockout	Causes albinism	-	Albino-phenotype mutants	Li G. et al. (2023)
*Cajanus cajan*	*CcPDS*	Knockout	Codes for phytoene dehydrogenase /Chromoplastic protein	CHOPCHOP	Albino phenotype with 15% transformation efficiency	Prasad et al. (2024)
*Cicer arietinum*	*CaPDS*	Knockout	Codes for phytoene dehydrogenase /Chromoplastic protein	CHOPCHOP	Mutants with albino phenotypes	Gupta et al. (2023)
	*4CL, RVE7*	Knockout	Drought tolerance	CHOPCHOP and CCTop	High efficiency in editing	Badhan et al. (2021)
	*CaSal1*	Base editing	Drought tolearnce	CHOPCHOP	Improved drough tolerance	Abdallah et al. (2025)
*Arachis Hypogaea* L	*AhFAD2A* and *AhFAD2B*	Targeted mutagenesis	Converts oleic acid to linoleic acid	CHOPCHOP	Increased ratio of oleic acid to linoleic acid in mutant plants	Rajyaguru and Tomar (2024)
	*AhPDS*	Knockout	Codes for phytoene dehydrogenase /Chromoplastic protein	CHOPCHOP	Albino phenotype with 20% transformation efficiency	Prasad et al. (2024)
	*AhALS*	Base editing	Targeted by over 50 commercial herbicides	-	Herbicide resistance in peanut	Shi et al. (2023)
	*AhNFR1* and *AhNFR5*	Knockout	Nodulation	CRISPR-P	Functional validation	Shu et al. (2020)
	*AhFAD2*	Knockout	Seed content improvement	CRISPR-P 1.0	*G448A, 441_442insA, G451T* mutations	Yuan et al. (2019)
*Vigna unguiculata*	*VuSPO11-1*	Knockout	Meiosis gene	-	Mutation induced	Che et al. (2021)
	*SPO11-1, REC8* and *OSD1*	Knockout	Meiosis genes	CRISPRdirect	Male and female sterilities	Juranić et al. (2020)
	*SNF*	Disruptive mutations	Symbiotic nitrogen fixation genes	CRISPR-P2.0	Blocked nodule formation	Ji et al. (2019)
*Glycine max*	*GmGLY1*	Knockout	Biosynthesis of glycitein (GLC)	CRISPR-P and CRISPR-PLANT	Decreased glycitein and increase daidzein content	Zhang P. et al. (2024)
	*GmFATA1* and *GmFATA2*	Frameshift mutations and knockouts	Encodes for oleoyl-ACP thioesterases	-	Increased beneficial fatty acid and oleic acid	Liao et al. (2024)
	*GmOMT*7	Knockout	Regulate the isoflavone content	http://crispr.hzau.edu Cn/CRISPR2/	Decreased isoflavone content in mutant plants	Yang et al. (2024)
	*lincRNA lincCG1*	Larger deletions	Regulate gene expression	CRISPR-P	*β*-conglycinin-deficient soybean lines	Song et al. (2024)
	*GmDMEa*	Knockout	Reduces seed size	Geneious software (v8.0)	Larger seeds and greater yields	Wang W. et al. (2024)
	*Tof4b*	Reading frame disruption	Delay flowering	-	Early flowering under long-day photoperiods	Fang et al. (2024)
	*GmCRY1*	Knockout	Regulate leaf senescence in response to blue light signals	CRISPRdirect	Show delayed leaf senescence and improve yield in fields	Li Z. et al. (2024)
	*GmSUT4.2*	Knockout	Sucrose transport	CRISPR-GE	Small leaf phenotype	Wu X. et al. (2024)
	*qFT13-3*	InDel	Downregulates the expression of *GmELF3b-2*	CRISPR-P	*qft13-3* mutants shorten the maturity period by 11 days	Li Y. F. et al. (2024)
	*GmDWF1a and GmDWF1b*	InDel	Brassinosteroid biosynthesis	CRISPR-P	Dwarf phenotype	Xiang et al. (2024)
	*SWEET11* and *SWEET21*	Knock out	Sucrose transport from the cotyledon to the hypocotyl	CRISPRdirect	Elongated hypocotyl phenotype	Su et al. (2024)
	*GmUVR8a, b, c, d*	Knock out	Receptor-perceived UV-B signal for Shoot-to-root communication	-	Leaf browning	Chen et al. (2024)
	*GmMATE100*	InDel	Import soyasaponins A and B into vacuoles	CRISPR-P 2.0	Decreased type-A and type-B soyasaponin contents roots	Ma et al. (2024)
	*MutL* homolog 1	Knock out	Meiotic crossover formation	-	Reduced pollen grain viability and increased embryo sac abortion	Wu T. et al. (2024)
	*GmNF-YC4*	Frameshift InDels	Delays flowering and maturation	CRISPR-P	Accelerated flowering and maturation under long day conditions	Cai et al. (2024)
	*GmCEP6*	InDel	Positively regulate nodulation	-	Significantly affected nodulation	Wu et al. (2024a)
	*J, JLa* and *JLb*	Frameshift indels	Regulate flowering time and increase yield in soybean	-	Delayed flowering phenotype	Li H. et al. (2024)
	*GmAP1d*	Knockout causing frameshift indels	Regulates flowering time under long-day photoperiods	CRISPRdirect	Flowering promoted under long-day conditions	Guo et al. (2024)
	*GmARM*	Knockout causing frameshift indels	Hormone response and stress regulation	-	Improved resistance to multiple stresses	Luo et al. (2024)
	*E4 gene*	Knockout	Photoperiodic flowering and maturity in soybean	CRISPR-P	Promotes maturation	Wu et al. (2024a)
	*GmHSP23.9*	Knockout	Regulate nodulation under elevated CO_2_ condition	CRISPR-P	Decrease in nodule number in response to elevated CO_2_ concentration	Niu et al. (2024)
	*GmFAD2*	Single base deletion and substitution	Oleic acid content	CRISPR-P	Creation of high oleic acid	Zhou et al. (2023)
	*GmPDCT*	Knockout	Regulation of oil synthesis	-	Creation of high oleic acid germplasm	Li H. B. et al. (2023)
	*GmSPL2b*	Knockout	Regulation of heat tolerance during flowering	CRISPR-P 2.0	Creation of heat-resistant varieties	Ding et al. (2023)
	*GmTAP1*	Knockout	Regulation of resistance to soybean blast	-	Creation of blast-resistant germplasm	Liu et al. (2023)
	*GmVPS8a*	Small indels	Regulation of phenotype	CRISPR-P	Verify that the gene is a multifunctional gene	Kong et al. (2023)
	*KAS1*	InDel mutations	Conversion of sucrose to oil	CRISPR-P	InDel mutations	Virdi et al. (2020)
	*GmFT2a and GmFT5a*	Knockout	Flowering time	CRISPR-P	*ft2a, ft5a*, and *ft2aft5a* mutants	Cai et al. (2020)
	*GmFAD2-1A, GmFAD2-2A*	Targeted mutagenesis	Peakoil biosynthesis	CRISPR-P	Increased oleic acid content	Wu et al. (2020)
	Pooled platform-102 candidate gene	Multiplex mutations	-	CRISPR-P	Multiplex mutations	Bai et al. (2020)
	*GmKIX8-1*	Loss-of-function mutagenesis	Regulates organ size	CCTop	Increased leaf and seed size	Nguyen et al. (2021)
	*FAD2-2*	Targated mutagenesis	Seed content improvement	CRISPR-P	Increased oleic acid content	Al Amin et al. (2019)
	*GmSPL9*	Knockout	Plant architecture	CRISPR-P	Altered plant architecture	Bao et al. (2019)
	*Glyma06g136900*	InDel mutations	Uncharacterized protein	-	InDel mutations	Di et al. (2019)
	*Glyma03g36470*	InDel mutations	Eukaryotic translation initiation factor	-	InDel mutations	Di et al. (2019)
	*Glyma14g04180*	InDel mutations	Late-embryogenesis abundant protein	-	InDel mutations	Di et al. (2019)
	*GmFAD2-1A, GmFAD2-1B*	Knockout using dual gRNA	Peakoil biosynthesis	CCTop	Increased oleic acid content	Do et al. (2019)
	*Conglycinin (7S)* and *glycinin (11S)*	Targeted knockout mutagenesis	Storage proteins	CRISPR-PLANT	Mutations in 3 of 9 genes	Li et al. (2019)
	*GmDrb2a* and *GmDrb2b*	Multiplexed targeting	Double-stranded RNA-binding2	Broad Institute’s sgRNA designer	Biallelic double mutant	Curtin et al. (2018)
	*GmFT2a and GmFT5a*	Deletion mutations	Flowering time	CRISPR-P	Deletion mutations	Cai Y. et al. (2018)
	*GmPDS11 and GmPDS18*	Targeted mutagenesis	Coding phytoene dehydrogenase/chromoplastic protei	CRISPR-P and CRISPR-PLANT	Albino and dwarf buds	Du et al. (2016)
	*Glyma06g14180*	Knockout	Uncharacterized protein	-	Targeted mutations created	Sun et al. (2015)
	*Glyma08g02290*	Knockout	Potassium transporter	-	Targeted mutations created	Sun et al. (2015)
	*Glyma12g37050*	Knockout	Ethylene receptor	-	Targeted mutations created	Sun et al. (2015)
	*GmFEI2*	Knockout to induce targeted indel mutations	LRR receptor-like serine/threonine-protein kinase FEI 2	CRISPR-P	Targeted mutations created	Cai et al. (2015)
	*GmSHR*	Knockout to induce targeted indel mutations	Short root protein	CRISPR-P	Targeted mutations created	Cai et al. (2015)
	*DD20* and *DD43*	Knockout	Two genomic sites on chromosome 4	-	Targeted mutations created	Li et al. (2015)
*Lotus japonicus*	*LYS6* and *LYS7*	Insertional mutation	Regulate arbuscular mycorrhiza	CRISPR-P	Reduced hyphal colonization and arbuscule formation	Fukuda et al. (2024)
	*Lbs genes*	Knockout	Nodule senescence	CRISPR-P	Early nodule senescence	Wang et al. (2019)
	*CYP716A51*	Frame-disrupting indels	Triterpenoid production	CRISPRdirect	Non-production of triterpenoids	Suzuki et al. (2019)
	*LjCZF1 and LjCZF2*	Frameshift mutations	Root nodule symbiosis	CRISPR-P	Decreased nodule formation	Cai K. K. et al. (2018)
	*SNF gene*	Knockout	Symbiotic nitrogen fixation	CRISPR-P	Mutation induced	Wang et al. (2016)

The use of dual gRNA (dgRNA) and multiplex gRNA strategies in legumes represent an emerging trend, although published reports remain limited. The dgRNA approach employs Cas9 Nickase guided by two gRNAs binding opposite strands, enabling targeted deletions (Jin and Marquardt, 2020; Song et al., 2024). In soybean, dgRNA-mediated editing has been successfully applied in several studies. Cai Y. et al. (2018) generated targeted deletions ranging from 599 to 1618 bp in *GmFT2a* and *GmFT5a,* with deletion frequencies of 15.6% and 15.8%, respectively. Do et al. (2019) edited the homeologous genes *GmFAD2–1A* and *GmFAD2–1B*, resulting in improved fatty acid composition. More recently, Song et al. (2024) used this strategy to develop β-conglycinin-deficient soybean lines, enhancing nutritional quality. Multiplex gRNA systems have also demonstrated considerable potential. Using the polycistronic tRNA–gRNA (PTG) method in *Medicago sativa*, Wolabu et al. (2020) achieved up to 75% mutation efficiency when targeting the stay-green gene (*MsSGR*), approximately 30-fold higher than single gRNA approaches. Subsequent multiplex editing of *MsFTa1* produced delayed-flowering mutants with a 78% and 76% increase in fresh weight and dry weight, respectively (Wolabu et al., 2023).

Effective gRNA design in legumes is further complicated by the fact that approximately 25% of legume species are polyploids or paleopolyploids, including alfalfa, peanut, and soybean (May et al., 2023). Polyploid genomes pose challenges beyond those encountered in diploid systems, including the need for conserved guides capable of targeting multiple alleles simultaneously, sub genome dominance, biased homeolog expression, and incomplete reference assemblies (Liu G. et al., 2020). When cultivar-specific genome sequences are unavailable, guides designed from reference genomes may fail due to SNP mismatches between the reference and target genotype, necessitating careful validation prior to experimental use (Saha et al., 2024). Incomplete assemblies may also overlook gene duplications or allelic variants, increasing the risk of missing important homologous targets (Zhang L. et al., 2021).

Finally, the efficacy of the selected guides can be tested *in vivo* (like protoplast transfection, hairy root transformation) or *in vitro* (sgRNA *In Vitro* Transcription and screening kit), before taking experiments at scale.

#### 2.3.4 CRISPR/Cas9 applications in legumes

CRISPR/Cas9 genome editing is now widely used in many legume species to improve both yield and quality traits, including oil composition, forage quality, and grain yield, *etc.* Applications of gene editing in major legumes *via* CRISPR/Cas9 is presented in Table 2. However, the commercialization of gene-edited crops requires regulatory approval under existing legislative and policy frameworks (Jones, 2015). Despite the growing number of reported applications, several biological and technical challenges continue to restrict the broader adoption of genome editing in legumes. Many legume species are recalcitrant to *in vitro* regeneration and transformation (Yadav et al., 2017). As a result, only certain tissues, such as immature cotyledons and mature cotyledons with embryonal axis, show ability to transform while others have capability of regeneration. Obtaining successful rooting is yet another obstacle. Several legumes lack stable regeneration protocol due to poor rooting, however efficient grafting techniques were reported (Das et al., 2017; Dewir et al., 2016). *Agrobacterium* mediated transformation is the primary method for transformation in grain legumes with few instances of biolistic transformation (Yadav et al., 2017; Das et al., 2017; Singh et al., 2022). Among the studied legumes, soybean stands out as the species with the most successful CRISPR/Cas9 applications (Bhowmik et al., 2021). Nevertheless, the use of CRISPR/Cas9 editing has potential to overcome the mentioned obstacles due to its precision, high efficiency and targeted editing of the gene of interest (Das and Acharjee, 2024).

##### 2.3.4.1 *M. truncatula* (Alfalfa)

*M. truncatula* serves as a key model legume because of its small, diploid genome, short life cycle, ability to reproduce by both selfing and outcrossing, production of indeterminate nodules, and the availability of a fully sequenced genome (Bottero et al., 2021). To validate genome editing efficiency and establish proof of concept, the *PHYTOENE DESATURASE* (*PDS*) gene was first targeted (Meng et al., 2017; Zhang et al., 2020). Trujillo et al. (2019) used multiplex CRISPR/Cas9 editing in *M, truncatula* to knock out up to five *NPD* (PLAT domain) genes. Mutants with multiple npd knockouts showed smaller, an early onset of nodule senescence or ineffective nodules, demonstrating that NPD proteins are essential for proper nodule development. Later, *MsSGR* gene was edited using CRISPR/Cas9, and the resulting mutants displayed altered coloration, which plays an important role in attracting insects and birds and may contribute to improved pollination (Wolabu et al., 2020). Researchers targeted *NOD26* gene to enhance the protein levels and generated plants with different doses of alleles (Bottero et al., 2021). In another study, *MtSUPERMAN* (*MtSUP*) gene was edited and mutants showed increased number of floral organs (disrupted floral architecture) (Rodas et al., 2021). Zhu et al. (2021) showed that while single and double MtCEP knockouts had no visible effects, triple (Mtcep1/2/12C) and quintuple (Mtcep1/2/5/8/12C) mutants developed more lateral roots and fewer nodules, demonstrating that MtCEP1, 2, and 12 redundantly regulate lateral root development and symbiotic nodulation. The knockout of the key *CYP450*gene of the non-hemolytic saponin pathway (*CYP93E2*), blocked the production of soya sapogenols in the leaves, stems and roots and redirected metabolic flux toward the accumulation of valuable hemolytic sapogenins (Confalonieri et al., 2021). In addition, editing *PHO2* gene using CRISPR/Cas9 led to phosphate hyperaccumulation in the mutants (Miller et al., 2022). More recently, Wolabu et al. (2023) used multiplex CRISPR/Cas9 to knock out all four copies of the flowering gene *MsFTa1*, producing plants that flowered later and accumulated substantially greater biomass, with increases of up to 78% and 76% fresh weight and dry weight, respectively as compared to control. These studies demonstrate the versatility of CRISPR/Cas9 in *M. truncatula*, supporting both tool optimization and functional analysis of key genes in nodulation, development, and secondary metabolism.

##### 2.3.4.2 L. japonicus

*L*. *japonicus* is also a key model legume that forms determinate nodules, like soybean and cowpea. CRISPR/Cas9 has been effectively applied for this crop, beginning with Wang et al. (2016), used hairy root transformation to mutate SNF (symbiotic nitrogen fixaton)-related genes. Cai et al. (2018a) edited cytokinin receptor Lotus histidine kinazI-interacting protein (LjCZF1), showing that its knockout reduced infection threads and nodules, confirming its role as a positive regulator of symbiotic nodulation. Wang et al. (2019) further demonstrated that loss of leghemoglobins (*Lbs*) causes early nodule senescence. Additionally, *CYP716A51* loss-of-function mutants revealed that this gene is required for producing C28-oxidized triterpenoids in *L. japonicus* hairy roots.

##### 2.3.4.3 *G. max L.* (Soybean)

Soybean is an important crop rich in protein and oil content and widely used for human and animal consumption (Freitas-Alves et al., 2025). The use of genome-editing tools has been accelerated to improve key agronomic traits in soybean (Bao et al., 2020). Initially, CRISPR studies were performed in hairy roots and protoplasts as low efficiency has been reported for stable transformation. Two endogenous genes (*GmFE12* and *GmSHR*) were targeted, and mutations (10%–93.3%) were detected in hairy roots (Cai et al., 2015; Li et al., 2015; Sun et al., 2015). Later, single gRNA was used to mutate *GmPDS*, *GmFAD2* (*Fatty Acid Desaturase 2*), *GmALS* (*Acetolactate Synthase*) genes (Zhang D. et al., 2021). Major advances have been made in flowering regulation, editing of *GmFT2a* and *GmFT5a* created late-flowering, heritable, transgene-free lines (Cai et al., 2020). Inactivation of *E1* leads to reduction in photoperiod sensitivity (Wan et al., 2022). Similarly, *GmPRR3b* plays a key role in flowering-time, the knockout of *gmPRR3bH6*, resulted in delayed growth and floral transition, whereas gmprr37-zgdd mutants flowered early under natural long-day conditions (Li et al., 2020; Wang et al., 2020). The improvement in seed quality has been achieved by knocking out *GmLox1/2/3* and eliminate lipoxygenase activity in mutants, in additions glycinin and conglycinin storage-protein genes has also been targeted (Li et al., 2019; Wang et al., 2020). Plant architecture has been modified by targeting *GmSPL9*, increasing node and branch numbers (Bao et al., 2019). Enhancing fatty-acid composition, *FAD2* family members have been targeted and achieved up to 95% efficiency and stable improvements across generations (Al Amin et al., 2019; Wu et al., 2020).

Large-scale CRISPR screens have identified several key regulators of soybean nodulation. The gmric1/gmric2 double mutants showed increased nodule production, whereas nodulation was reduced in gmrdn1 triple mutants (Bai et al., 2020). In another set of studies, optimization of sgRNA expression using soybean U6 promoters improved CRISPR/Cas9 editing efficiency (Di et al., 2019), while egg cell–specific promoter CRISPR/Cas9 systems enabled efficient recovery of heritable mutations in soybean, including edits in reporter and endogenous genes (Zheng et al., 2020). The same strategy also produced high-frequency knockout and in-frame alleles of the oil-biosynthesis gene *KASI* (Virdi et al., 2020). Taken together, these advances position soybean as one of the most actively edited legume crops, however, wider application will still require further gains in stable transformation efficiency. Moreover, most soybean genome-editing efforts continue to rely on Cas9, nCas9-based base editors, and Cas12a nucleases (Freitas-Alves et al., 2025).

##### 2.3.4.4 *V*. *unguiculata* (Cowpea)

Cowpea is a nutritionally important legume valued for its high protein content and associated health benefits. It also exhibits strong symbiotic nitrogen fixation (SNF) capacity, resilience under low-rainfall conditions, and comparatively low fertilizer requirements. Owing to these agronomically important traits, cowpea has become one of the most widely cultivated and economically significant legumes worldwide (Ji et al., 2019; Che et al., 2021), thereby motivating increasing efforts to apply gene-editing technologies for crop improvement. The first demonstration of CRISPR/Cas9-mediated genome editing in cowpea targeted SNF-related genes using a transgenic hairy-root system (Ji et al., 2019). Disruption of both alleles completely abolished nodule formation, although the induced mutations were not inherited, confirming the functional relevance of these genes. Subsequently, meiosis-associated genes, including *SPO11-1*, *REC8,* and *OSD1*, were targeted to explore reproductive manipulation. Biallelic mutations in exon 1 and exon 3 of *SPO11-1* disrupted meiosis and resulted in complete male and female sterility in T0 plants, underscoring the essential roles of these reproductive genes (Juranić et al., 2020). More recently, Che et al. (2021) targeted the *VuSPO11-1* using CRISPR/Cas9 and reported efficient editing at the intended target site. A transient leaf assay was developed to assess genome-editing constructs prior to stable cowpea transformation, substantially streamlining the editing pipeline and accelerating improvement programs.

Together, these studies establish a foundation for functional-genomics and gene-editing applications in cowpea and highlight the potential of CRISPR-based tools to accelerate genetic improvement in this important legume.

##### 2.3.4.5 *C*. *arietinum* L. (Chickpea)

Chickpea is an important legume crop, particularly among vegetarian populations, because it is a rich source of protein. Globally, India ranks first in chickpea production (Mishra, 2025). However, chickpea yield is strongly constrained by a range of biotic and abiotic stresses, with drought and extreme temperatures being among the most critical. Recent studies have demonstrated the potential of CRISPR/Cas9 for improving stress resilience in chickpea. For example, targeted editing of stress-related genes such as *4-coumarate ligase* (*4CL*) and *Reveille 7* (*RVE7*) in chickpea protoplasts has provided important insights into the molecular mechanisms underlying drought tolerance (Badhan et al., 2021). Similarly, casal1 mutants generated through CRISPR/Cas9 exhibited enhanced drought tolerance and altered root architecture, enabling plants to better withstand water stress (Abdallah et al., 2025). Collectively, these findings highlight CRISPR technology as a promising tool for developing chickpea varieties with improved tolerance to drought and heat stress.

##### 2.3.4.6 *A*. *hypogaea* (Peanuts or groundnuts)

Groundnut (peanut) is a major legume crop valued for its high oleic acid content, and improving oil quality and content remains a key breeding objective due to its industrial importance, extended shelf life, and antioxidant properties. CRISPR/Cas9-based site-specific genome editing has been successfully used to modify fatty acid biosynthesis to improve oil quality. Neelakandan et al. (2022b) reduced the seed-specific expression of two homologous *FAD2* genes while preserving their function in other tissues, leading to increased oleic acid content. In peanut, the genes *AhFAD2A* and *AhFAD2B* encode the FAD2 enzyme responsible for converting oleic acid to linoleic acid, and the Indian cultivar GG20 lacks natural mutations in *AhFAD2B*. Targeted editing of the *AhFAD2B* gene, which catalyzes the conversion of oleic acid to linoleic acid, was successfully achieved using CRISPR/Cas9 in groundnut protoplast cultures, leading to desirable alterations in fatty acid composition (Rajyaguru and Tomar, 2024). In addition, editing of *AhFatB* using CRISPR/Cas9 altered the fatty acid profile by reducing palmitic acid and increasing oleic acid levels (Tang et al., 2022). These CRISPR-derived peanut mutants with modified saturated fatty acid content show strong potential for producing healthier, higher-quality peanut oil. Beyond oil quality, CRISPR/Cas9 has also been applied to improve peanut nutritional and agronomic traits. Editing of the major allergen gene *Arah2*, produced lines with enhanced nutritional suitability for individuals with peanut allergies. Furthermore, Shu et al. (2020) demonstrated the utility of CRISPR/Cas9 in functional genomics by dissecting the role of Nod factor receptors (NREs) in peanut nodulation. Mutants lacking both *AhNFR5* genes displayed a Nod^−^ phenotype, whereas plants edited for *AhNFR1* genes retained the ability to form nodules after rhizobial inoculation. Together, these studies highlight the versatility of CRISPR/Cas9 for improving oil quality, nutritional safety, and symbiotic nitrogen fixation.

##### 2.3.4.7 *P. sativum* (Pea)

Lipoxygenase (LOX) enzymes are abundant in seeds and generate volatile organic compounds from polyunsaturated fatty acids, often causing undesirable flavors in legumes such as pea and soybean. CRISPR/Cas9 mediated mutagenesis of *PsLOX2* significantly improved seed aroma and fatty acid composition in an elite Canadian pea variety (Bhowmik et al., 2023). In addition, saponins glycosylated triterpenoids responsible for bitter and astringent tastes in pea products were effectively reduced through CRISPR/Cas9 knockout of *β-amyrin synthase* (*PsBAS1*), a key enzyme initiating saponin biosynthesis. Targeted editing produced bi-allelic psbas1 mutants with a 99.8% reduction in seed saponins, along with a modest increase in protein content and a slight decrease in starch (Hodgins et al., 2024). Together, these studies demonstrate that CRISPR/Cas9 enables precise, efficient improvement of pea flavor and nutritional quality across cultivars.

### 2.4 Base editing (BE)

BE represents another CRISPR-derived technology that enables precise nucleotide conversions without generating DSBs. DNA base editors consist of a catalytically impaired Cas nuclease fused to a nucleobase deaminase, and in some configurations, a DNA glycosylase inhibitor. This system directly converts one nucleotide or base pair to another, minimizing unwanted by-products (Rees and Liu, 2018). Several BE systems have been developed to date, including the two foundational classes, cytosine base editors (CBEs), which convert C•G to T•A, and adenine base editors (ABEs), which convert A•T to G•C, as well as more recently engineered systems such as cytosine and guanine base editors (CGBEs), which convert C•G to G•C and dual base editors capable of simultaneous C to T and A to G conversions within a single editing window (Komor et al., 2016; Gaudelli et al., 2017; Grünewald et al., 2019; Zhao et al., 2021). The feasibility of BE in legumes has now been demonstrated across several species. In soybean, Cai et al. (2020) introduced targeted single-base substitutions in *GmFT2a* and *GmFT4*, while CBE was used in peanut to modify *AhFAD2A* and *AhFAD2B*, which encode fatty acid desaturase 2 (FAD2), a key enzyme converting oleic acid to linoleic acid (Neelakandan et al., 2022a). More recently, Bai et al. (2023) engineered a high-efficiency CBE by coupling PmCDA1 with a GmRad51 DNA-binding domain, achieving editing efficiencies up to 52.6% and generating plants with the expected non-nodulating phenotype. Advances have also extended to PAM-relaxed systems. Chen et al. (2024) reported the application of CRISPR-SpRY in soybean, demonstrating efficient mutagenesis at non-canonical PAM sites. Both the SpRY-based cytosine editor (SpRY-hA3A) and adenine editor (SpRY-ABE8e) enabled precise C to T and A to G conversions, substantially expanding the accessible target space for soybean genome editing. BE in legumes is constrained by low transformation efficiency, strong recalcitrance to plant regeneration, and limited PAM site availability, which together restrict successful delivery and targeting of editing reagents. Achieving transgene-free edited plants remains particularly difficult in legumes, often requiring multiple generations of segregation, which slows deployment and limits practical adoption (Baloglu al., 2022).

### 2.5 Prime editing (PE)

PE, first described by Anzalone et al. (2019), represents a versatile and precise genome editing platform. In this system, a Cas9 nickase that introduces a single-strand break is fused to a reverse transcriptase, which synthesizes new DNA using a PE gRNA (pegRNA) as a template. The pegRNA contains a spacer and scaffold region, together with a reverse-transcription template (RTT) carrying the desired edit and a prime-binding site (PBS). PE enables highly accurate sequence modification and is generally less error-prone than conventional CRISPR/Cas systems. It can introduce specific single-base substitutions, supports all 12 possible nucleotide conversions, and can generate targeted insertions and deletions without the need for donor DNA (Mikhaylova et al., 2024). Despite its promise, application of PE in legumes remains limited. To date, only one study has reported its use. Biswas et al. (2022) optimized PE in protoplasts of peanut, chickpea, and cowpea by restoring GFP activity, achieving relatively low editing efficiencies (0.2%–0.5%). PE in plants generally shows lower efficiency compared to standard CRISPR/Cas9, often requiring extensive, time-consuming optimization of the PE gRNA (pegRNA) for each specific target. In legume studies, PE adoption remains limited due to significant biological and technical constraints, primarily characterized by low editing efficiency and high recalcitrance to transformation (Maia et al., 2024). Editing efficiency can be extremely low, sometimes reported between 0.2% and 0.5% in protoplasts, far lower than in rice (Asiamah et al., 2025).

Overall, the application of gene editing techniques for legume improvement is accompanied by both technical and policy-related constraints. From a technical perspective, precision remains a central concern. Although CRISPR/Cas9 is designed to introduce targeted modifications, unintended alterations may occur at genomic sites that share partial sequence homology with the intended target. These off-target events can result in unexpected mutations, thereby raising questions about reliability, biosafety, and long-term genetic stability. In addition to technical limitations, regulatory framework across countries significantly influences the development and commercialization of genome-edited crops. The governance of gene-editing technologies differs markedly among jurisdictions, creating uncertainty for breeders, researchers, and seed companies operating in global markets (Singer et al., 2024). Regulatory framework also directly affects the role of gene editing in addressing food security challenges. The ability to rapidly deploy legumes with enhanced tolerance to biotic and abiotic stresses, improved nutritional profiles, or increased productivity depends largely on clear and predictable approval pathways. Regulation of gene-edited crops varies widely worldwide. The European Union and New Zealand apply strict Genetically Modified Organism (GMO) regulations, whereas countries such as the United States, Colombia, Canada, and Argentina follow product-based approaches. Similar policies are used in few Asian countries (India, China, Japan) and Australia (Rose et al., 2020; Asiamah et al., 2025; Thakur et al., 2025). In many countries, uncertainty remains regarding how genome-edited crops should be regulated, partly due to the rapidly evolving nature of genome-editing technologies and inconsistent use of terminology (Pixley et al., 2022). Overcoming current regulatory challenges for gene-edited crops will require coordinated efforts from researchers, investors, policymakers, and consumers. Genome editing may involve the temporary introduction of foreign DNA, may or may not result in transgenic products, and can generate plant varieties that are indistinguishable from those developed through conventional breeding (Pixley et al., 2012). These complexities make the development of clear, science-based regulatory frameworks essential. Researchers can contribute by developing more precise genome-editing technologies with reduced off-target effects and by participating in policy discussions that help establish evidence-based regulations capable of distinguishing gene-edited crops from transgenic crops (Scheben and Edwards, 2018). Investors and funding agencies also play an important role by supporting research and innovation programs that consider regulatory requirements and market needs from the outset, thereby facilitating the successful development and deployment of improved crop varieties (Pixley et al., 2022). In addition, transparent communication and public engagement are essential to inform consumers about the benefits, safety considerations, and ethical aspects of gene-edited crops, helping to build public trust and support informed regulatory decision-making (Lassoued et al., 2021; Mbaya et al., 2022). Gene editing technologies have potential to produce more resilient, nutritious, and high-yielding crops, especially improved legumes, to help meet growing demands for plant-based protein and address climate and disease pressures. Harmonized global regulations would promote responsible use, facilitate trade, and enable these crops to contribute more effectively to sustainable agriculture.

## 3 Conclusion and future perspectives

Gene editing is rapidly evolving from a gene-disruption tool into a precision breeding platform with significant potential for legume improvement. However, realizing this potential requires addressing the key challenges that currently limit genome editing progress in legumes. Among these challenges, efficient plant transformation and regeneration remains the most critical. The successful outcome of gene editing technologies largely rely on the development of effective pipelines and protocols for transformation and regeneration in legume crops supported by detailed molecular characterization in line with the guidelines laid by regulatory authority. Advances in transformation and regeneration technologies, including transient expression systems, morphogenic regulators, and DNA-free editing approaches, are expected to overcome long-standing genotype dependence and expand gene editing to recalcitrant and underutilized legume species. Beyond transformation barriers, the next phase of legume improvement will increasingly rely on the deployment of BE and PE, and the newly emerging third generation of CRISPR technologies that can address some of the challenges of earlier generations (Gupta and Kumar, 2025). The continued success of genome editing in legumes will also depend on improved functional understanding of gene networks and trait regulation. Expanding omics resources, including pan-genomes, transcriptomes, and epigenomics, will support the identification of high value editing targets. Emerging approaches such as *de novo* domestication of crop wild relatives (CWRs) and the reintroduction of beneficial alleles through re-wilding strategies provide additional opportunities to generate novel diversity that can be refined through genome editing (Cardi et al., 2023). Research especially in legumes with complex epigenomes shall unlock the full potential of epigenome engineering (Leech et al., 2026).

Major research areas for engineering in legumes are evolving with climate variability with new challenges emerging. Emphasis of higher productivity and improvement of related agronomic traits including plant ideotype, nutrient use-efficiency, machine harvestable genotypes, herbicide-tolerance, climate resilient-traits, disease resistance or tolerance, abiotic stress enhancement, nutritional enrichment and legume–rhizobia interactions are some of the crucial areas. Targeted editing of metabolic pathways (Synthetic Biology) will enable the development of nutrient-dense legumes with reduced anti-nutritional factors, supporting global food nutritional security and sustainability. Niche-specific basic and strategic research needs to be conducted to understand the biochemical basis of the phenotype and generation of omics resources to support the understanding. We advocate use of elite genotypes largely grown by legume farmers and starting genotype for improvement programs.

Looking ahead, the integration of gene editing with pan-genomics, artificial intelligence-assisted target discovery, high-throughput phenotyping and phenomics, and speed breeding will dramatically shorten breeding cycles and improve genetic gain in legumes. Software for designing intuitive gRNA is growing rapidly and newer software versions in synergy with genome sequence information are more efficient and user-friendly. Recently, Artificial Intelligence (AI) enabled design using large language models trained on biological diversity at scale presents a powerful alternative with the potential to generate editors with optimal properties for targeted species, bypassing evolutionary constraints of prokaryotic based editors (Ruffolo et al., 2025).

Ultimately, the successful deployment of gene editing in legumes will depend not only on technological innovation but also on enabling regulatory frameworks and equitable access to genomic resources and editing tools. The development of transgene-free editing approaches may facilitate broader regulatory acceptance and accelerate field deployment. Coordinated progress in technology development, regulatory harmonization, and capacity building in legume-producing regions will be essential to ensure that genome editing contributes effectively to global food security, sustainable agriculture, and resilient cropping systems.
