# Supplementary material for: Advances in gene editing for legume improvement: technologies, progress, and prospects
Source: Front Genome Ed. 2026 Apr 15;8:1789952. doi: 10.3389/fgeed.2026.1789952 (PMC13126311; doi:10.3389/fgeed.2026.1789952)
Supplement: Supplementary file 1 [file Table1.docx]

Table S1. Key limitations of the gRNA prediction and designing tools

| **Tool** | **Key Limitations** | **References** |
| --- | --- | --- |
| **CHOPCHOP** | 1.Off-target prediction relies on sequence alignment  2. Misses bulges/complex mismatches.  3. Efficiency scoring uses older models  4. Ignores chromatin/epigenetic context. | Labun et al., 2019; Haeussler et al., 2016 |
| **CRISPR-P 2.0** | 1. Plant-specific  2. Limited to well-annotated genomes.  3. Simple mismatch-based off-target prediction  4.Lacks advanced scoring (CFD/deep learning). | Liu et al., 2017 |
| **CRISPR-PLANT** | 1. Applicable to plants only.  2. Simplified off-target and efficiency scoring  3. Limited transferability. | Lei et al., 2014 |
| **Benchling** | 1. Limited transparency of scoring integration.  2. Advanced off-target analysis/batch processing often requires paid subscription.  3. Minimal bulge/chromatin modeling  4. Scoring varies across tools. | Haeussler et al., 2016; Tycko et al., 2019 |
| **sgRNAcas9** | 1. Outdated heuristics  2. Lacks modern ML scoring.  3. Minimal off-target context  4. Command-line interface reduces accessibility. | Xie et al., 2014 |
| **CRISPRdirect** | 1. Simple mismatch/seed-based filtering only.  2. No on-target efficiency scoring  3. Limited genome support. | Naito et al., 2015 |
| **Cas-OFFinder** | 1. Off-target identification only  2. No guide design or efficiency prediction.  3. Outputs unranked  4. Ignores chromatin/expression context. | Bae et al., 2014; Tycko et al., 2019 |
| **E-CRISP v5.4** | 1. Traditional mismatch-based off-target prediction.  2. Old efficiency scoring system.  3. Limited batch processing.  4. No epigenomic/contextual scoring. | Heigwer et al., 2014 |
| **CRISPOR** | 1. Computationally intensive for large regions.  2. Accuracy depends on reference genome | Haeussler et al., 2016 |
| **CRISPseek** | 1. Simple mismatch-based algorithm.  2. No machine-learning or genomic context integration. | Zhu et al., 2014 |

References

Bae, S., Park, J., & Kim, J. (2014). Cas-OFFinder: a fast and versatile algorithm that searches for potential off-target sites of Cas9 RNA-guided endonucleases. *Bioinformatics*, 30(10),1473-1475. https://doi.org/10.1093/bioinformatics/btu048

Haeussler, M., Schönig, K., Eckert, H., Eschstruth, A., Mianné, J., Renaud, J.B., Concordet, J.-P. (2016). Evaluation of off-target and on-target scoring algorithms and integration into the guide RNA selection tool CRISPOR. *Genome Biol.* 17, 148. doi:10.1186/s13059-016-1012-2

Heigwer, F., Kerr, G., & Boutros, M. (2014). E-CRISP: fast CRISPR target site identification. *Nat. Methods* 11(2), 122-123.  doi.org/10.1038/nmeth.2812

Labun, K., Montague, T. G., Krause, M., Torres Cleuren, Y. N., Tjeldnes, H., & Valen, E. (2019). CHOPCHOP v3: Expanding the CRISPR web toolbox beyond genome editing. *Nucleic Acids Res.* 47(W1), W171–W174. doi:10.1093/nar/gkz365

Lei, Y., Lu, L., Liu, H.-Y., Li, S., Xing, F., & Chen, L.L. (2014). CRISPR-P: A web tool for synthetic single-guide RNA design of CRISPR-system in plants. *Bioinformatics* 30(18), 2676–2677. doi.org/10.1093/bioinformatics/btu445

Liu, H., Ding, Y., Zhou, Y., Jin, W., Xie, K., & Chen, L.L. (2017). CRISPR-P 2.0: An improved CRISPR-Cas9 tool for genome editing in plants. *Mol. Plant* 10(3), 530–532. https://doi.org/10.1016/j.molp.2017.01.003

Naito, Y., Hino, K., Bono, H., & Ui-Tei, K. (2015). CRISPRdirect: Software for designing CRISPR/Cas guide RNA with reduced off-target sites. *Bioinformatics* 31(7), 1120–1123. doi: 10.1093/bioinformatics/btu743

Tycko, J., Myer, V. E., & Hsu, P. D. (2019). Methods for optimizing CRISPR-Cas9 genome editing specificity. *Nat Rev Genet.* 20, 515–529. doi.org/10.1038/s41576-019-0137-0

Xie, S., Shen, B., Zhang, C., Huang, X., & Zhang, Y. (2014). SGRNACAS9: A software package for designing CRISPR SGRNA and evaluating potential Off-Target cleavage sites. PLoS ONE, 9(6), e100448. doi.org/10.1371/journal.pone.0100448

Zhu, L. J., Holmes, B. R., Aronin, N., & Brodsky, M. H. (2014). CRISPRSeek: a bioconductor package to identify Target-Specific guide RNAs for CRISPR-CAS9. Genome-Editing systems. PLoSONE, 9(9), e108424. doi.org/10.1371/journal.pone.0108424
